# Refined cottonseed oil as a replacement for soybean oil in broiler diet

**DOI:** 10.1002/fsn3.933

**Published:** 2019-02-05

**Authors:** Ao Yang, Ming Qi, Xu Wang, Shuai Wang, Lvhui Sun, Desheng Qi, Luoyi Zhu, Yongzhi Duan, Xin Gao, Shahid Ali Rajput, Niya Zhang

**Affiliations:** ^1^ Department of Animal Nutrition and Feed Science College of Animal Science and Technology Huazhong Agricultural University Wuhan China; ^2^ Hunan Provincial Engineering Research Center for Healthy Livestock and Poultry Production Key Laboratory of Agro‐ecological Processes in Subtropical Region Institute of Subtropical Agriculture Chinese Academy of Sciences Changsha China; ^3^ University of the Chinese Academy of Sciences Beijing China

**Keywords:** antioxidant status, broiler, cottonseed oil, cyclopropenoid fatty acids, fatty acid composition, performance

## Abstract

With the shortage of common vegetable fat sources, such as soybean oil (SBO), it is urgent to find alternative oil sources for broiler producers. The objective of this study was to evaluate the potential of refined cottonseed oil (CSO) as a replacement for SBO in broiler diets. A total of 180 chickens at 1 d of age were randomly assigned to five treatments of six replicates. One treatment was the basal diet (control), and the other four experimental diets were formulated from the basal diet by replacing (w/w) 25%, 50%, 75%, and 100% of the SBO with refined CSO (only containing 0.2% cyclopropenoid fatty acids, and no free gossypol was detected). At the end of week 6, blood samples were obtained from the jugular vein and the breast muscle was aseptically isolated from two birds per replicate. The results showed that substitution of CSO for low‐level SBO had no significant effect (*p *>* *0.05) on broiler performance during the starter period (week 1–3), while 50% level of CSO inclusion significantly increased (*p *<* *0.05) ADG and improved FCR compared with the control group during the finisher period (week 4–6). Broilers fed 100% CSO diets had lower (*p *<* *0.05) levels of serum total protein (TP), albumin (ALB), cholesterol (CHO) concentrations, and serum alkaline phosphatase (AKP) activity than that of the control broilers. Furthermore, the serum antioxidant status appeared to be enhanced by CSO. Additionally, high levels of CSO (75 and 100%) significantly increased the proportions of C14:0 and C18:0 but decreased the proportions of C18:1n9t, C18:2n6c, and ∑ n‐6 polyunsaturated fatty acids in breast muscles of broilers. Overall, the SBO could be replaced with refined CSO up to 50% in diets for broilers without adversely affecting the performance, liver functions, and breast muscle fatty acid composition of these broilers.

## 1 INTRODUCTION

Poultry meat is often preferred over other meats mainly due to its low lipid content and relatively high content of polyunsaturated fatty acids (PUFA) (Nkukwana et al., 2014). Fatty acid profile of poultry body is deeply affected by the dietary fatty acid profile (Rymer & Givens, 2005). To meet the recommended energy requirement of broilers, generally it is routine to supplement fats and oils in commercial broiler diets. Therefore, the fats and oils supplemented in the diet are crucial to the meat fatty acid composition. Vegetable oils, rich in unsaturated fatty acids (UFAs), have higher metabolizable energy for poultry than saturated fatty acids (SFAs) from animal fats (Crespo & Esteve‐Garcia, 2001). However, the decreasing availability of common vegetable fat sources, such as soybean oil (SBO) and corn oil, makes it urgent for broiler producers to look for alternate oils sources.

Cotton is an essential oil crop in China. The cottonseed production in China has ranked first in the world over the years. For example, the cottonseed production in China was 13,680,000 metric tons in 2012 (FAO, 2013), and the yield of cottonseed oil (CSO) was estimated to be 2,052,000 metric tons. It is indicated that CSO is an easily accessible vegetable oil source for broiler production. Cottonseed oil possesses high metabolizable energy for broiler, which is similar to that of soya oil. Additionally, the concentrations of unsaturated fatty acids in CSO are relatively high. Cottonseed oil has a fatty acid profile that composed of 52.89% linoleic acid, 25.39% palmitic acid, 16.35% oleic acids, together with small amounts of 2.33% stearic acid, 1% myristic acid, 0.6% palmitoleic acid as well as 0.17% linolenic acid (Radcliffe, Czajka‐Narins, & Imrhan, 2004). Hence, CSO has been considered a promising alternative to SBO or corn oil in the broiler production. However, the high concentrations of free gossypol (FG) and cyclopropenoid fatty acids (CPFAs, including malvalic acid and sterculic acid) arouse public concern about its adverse effects on poultry production.

Free gossypol is a main factor restrainting the application of cottonseed products in feed, and high concentrations of free gossypol may cause various acute clinical signals of poisoning, including respiratory distress, reduction in body weight gain, anorexia and weakness. (Gadelha, Fonseca, Oloris, Melo, & Soto‐Blanco, 2014). There have been numerous studies carried out to evaluate the effects of different concentrations of free gossypol contained in diets on the performance and physiological status of broiler chickens (Mishra, Ray, Sarkar, & Haldar, 2015; Özdoğan, Wellmann, & Paksuz, 2012; Pieniazek et al., 2015), and also many treatment methods having been explored to eliminate the adverse influences of free gossypol, including ferrous treatment (Aguiar et al., 2016), microbial treatment (Khalaf & Meleigy, 2008), ammonia treatment (Vohra, Hafez, Earl, & Kratzer, 1975), and so on. Meanwhile, cyclopropenoid fatty acids, including malvalic acid and sterculic acid, are special fatty acids that can be found from oilseeds in the order malvales (Phelps, Shenstone, Kemmerer, & Evans, 1965; Ralaimanarivo, Gaydou, & Bianchini, 1982; Vickery, 1980), and their presence in cottonseed oil has caused many deleterious biological effects when fed to animals, such as pink white and associated defects in eggs, depression of egg production, and so on (Phelps et al., 1965; Shenstone, Vickery, & Johnson, 1965). Among these biological effects on animals, the most significant effect it caused must be altering fatty acid profile in animal tissues. It has been reported that the cattle fed CSO for 70 to 80 days has more C18:0 SFA and less C16:1 and C18:1 UFAs in adipose tissue (Yang, Larsen, Smith, & Tume, 1999). Radcliffe et al. (2004) found that the levels of SFAs in adipose tissue of mice were much higher for the CSO group than the control, together with lower levels of monounsaturated fatty acids (MUFAs) and n‐3 PUFAs. Evans et al. also observed that eggs, blood plasma, and tissues from hens fed a diet that contained crude cottonseed oil contained a higher proportion of stearic acid and a lower proportion of oleic acid than that from hens fed the normal diet (Evans, Bandemer, Anderson, & Davidson, 1962; Evans, Davidson, & Bandemer, 1961; Evans, Flegal, Foerder, Bauer, & La Vigne, 1977). It is well known that higher concentrations of SFAs in diet would increase the atherogenic lipid profiles (Matsumori et al., 2013) and serum cholesterol of consumer (Grundy & Vega, 1988). However, Radcliffe and Czajka‐Narins found that replacement of dietary corn oil with CSO brings about a decrease of serum total cholesterol and high‐density lipoprotein (HDL) for male rats (Radcliffe & Czajka‐Narins, 2006). These separate findings are somewhat confusing.

The inclusion of cottonseed meal in feed for birds has been studied for decades (El Boushy & Raterink, 1989). However, little research has been performed to evaluate the effects of CSO in broilers, especially the effects of CSO containing CPFAs on broilers’ health status and fatty acid profile. In the present study, we investigated the effects of refined CSO dietary supplementation on growth performance, serum biochemistry, and antioxidant status, as well as fatty acid profile of breast muscle in broilers. Then, we looked forward to explore which adverse effects would be caused on the broilers when fed CSO contained CPFAs and evaluated the potential of refined CSO as a replacement for SBO in broiler diets.

## 2 MATERIALS AND METHODS

### 2.1 Birds, experimental design, and sample collection

All animal procedures used in this study were approved by the Institutional Animal Care and Use Committee of Huazhong Agricultural University, China. A total of 180 1‐day‐old Cobb 500 male broiler chickens were randomly allocated into five treatments consisting of six replicates with six birds per replicate. The chickens were housed in stainless steel cages with a 16 hr/day illumination cycle (10 to 20 Lx) and had ad libitum access to feed and water. The basal diets were formulated to meet the nutritional requirements of broilers (NRC, 1994, Table 1). The CSO was purchased from commercial oil plant and had been refined and processed before transported out of the plant. The fatty acid profile of CSO and SBO was determined in our laboratory using GC (Table 2), and the processing of oils was same as described below. No free gossypol was detected in oils, the detection of free gossypol was subjected to HPLC, and the method was described in our previous studies (Zhu et al., 2018). Four experimental diets were formulated from the starter and finisher basal diets by replacing (w/w) 25%, 50%, 75%, and 100% of the SBO with CSO. After 3 days of acclimation, broilers in each treatment were fed the corresponding diet for 6 week. The mortality of birds was monitored daily, whereas body weights and feed intake were measured triweekly. At the end of week 6, two birds per replicate were randomly selected, euthanized, and sampled. Blood samples were collected from the jugular vein into uncoated serum tubes and then centrifuged at 3,000 *g* for 10 min at 4°C to obtain serum. The serum samples were stored at −20°C until analysis. The breast muscle was aseptically isolated, flushed with ice‐cold isotonic saline and then immediately immersing them in liquid nitrogen and stored at −80°C for later fatty acids analysis.

**Table 1:** Composition of basal diets and nutrient levels^a^

Ingredients	Percentage (%)		Nutrient level	Content	
	1–3 week	4–6 week		1–3 week	4–6 week
Maize	60.38	58.41	ME (kcal/kg)	2,924.90	3,099.90
Dehulled soybean meal	28.47	25.90	Crude protein (%)	21.01	19.01
DDGS	4.00	3.93	Calcium (%)	0.90	0.75
Maize gluten	2.33	0.00	Total phosphorus (%)	0.65	0.59
Wheat	0.00	5.00	Available phosphorus (%)	0.40	0.32
Calcium hydrophosphate	1.58	1.08	Methione (%)	0.50	0.46
Limestone	2.00	2.00	Lysine (%)	1.25	1.15
Soybean oil	0.50	3.05	Salt	0.23	0.23
Premix^b^	0.50	0.50			
Salt	0.20	0.20			
Lysine (70%)	0.46	0.42			
DL‐Methionine	0.27	0.28			
Threonine (98.5%)	0.10	0.10			
Sodium bicarbonate	0.10	0.10			

^a^The experimental diets were formulated from the basal diets by replacing (w/w) 25%, 50%, 75%, and 100% soybean oil with cottonseed oil. ^b^The premix provided the following per kg of diet: (1–3 week) vitamin A, 9,500 IU; cholecalciferol, 3,600 IU; vitamin E, 50 mg; thiamine, 3.6 mg; riboflavin, 10 mg; pyridoxine, 5.5 mg; iron, 100 mg; manganese, 110 mg; copper, 10 mg; zinc, 100 mg; selenium, 0.4 mg; iodine, 0.3 mg; (4–6 week) vitamin A, 15,000 IU; cholecalciferol 4,000 IU; vitamin E, 50 mg; thiamine, 3.60 mg; riboflavin, 10 mg; pyridoxine, 5.5 mg; iron, 100 mg; manganese, 110 mg; copper, 10 mg; zinc, 100 mg; selenium, 0.4 mg; and iodine, 0.3 mg.

**Table 2:** The fatty acid composition of soybean oil and cottonseed oil (g/100 g of total fatty acids)

Fatty acid	Soybean oil	Cottonseed oil
C16:0	8.05	19.78
C17:0	0.05	0.06
C18:0	2.84	1.57
C20:0	2.52	1.25
C22:0	0.20	0.15
C24:0	0.07	0.05
C16:1	0.05	0.40
C18:1n9t	0.20	0.02
C18:1n9c	17.45	15.59
C18:2n6c	52.23	54.50
C18:3n6	0.43	0.17
C18:3n3	8.09	0.75
C20:2	0.30	0.18
C18:1 cpe^a^	ND	0.12
C19:1 cpe	ND	0.08

Notes

ND: not detected.

a C18:1 cpe, malvalic acid; C19:1 cpe, sterculic acid.

### 2.2 Serum biochemistry assays

The activities of alanine aminotransferase (ALT), aspartate aminotransferase (AST), alkaline phosphatase (AKP), and gamma‐glutamyl transpeptidase (GGT), along the concentrations of total protein (TP), albumin (ALB), cholesterol (CHO), triglycerides (TG), high‐density lipoprotein cholesterol (HDL‐C), and low‐density lipoprotein cholesterol (LDL‐C), in serum were determined by corresponding commercial kits (DiaSys Diagnostic System, Shanghai, China) using automatic biochemical analyzer (Beckman Synchron CX4 PRO. CA, USA).

### 2.3 Assay of serum antioxidant enzyme activities

The antioxidant indices including glutathione peroxide (GPX), glutathione S‐Transferases (GST), total antioxidant capacity (T‐AOC), superoxide dismutase (SOD) activities, and malondialdehyde (MDA) content in serum were measured using commercial kits (Nanjing Jiancheng Bioengineering Institute, Nanjing, China) according to the manufacturer's instructions.

### 2.4 Determination of fatty acids profiles of breast muscle

The total fatty acids were extracted from the muscle based on the method of Folch, Lees, and Sloane Stanley (1957) and were methylated according to Slover and Lanza (1979). The composition of total fatty acids in the breast muscle was determined using a Shimadzu 2010 plus gas chromatograph equipped with a capillary column (SPTM‐2560, 100 m × 0.25 mm ID, 0.20 μm film, Supelco, Bellafonte, PA, USA). The initial temperature of the oven was 140°C and was increased by 4°C/min to 240°C. The temperature of the injector and the flame ionization detector was kept at 260°C. Nitrogen was used as the carrier gas at a flow rate of 2 cm/s with an injection split ratio of 100:1 (v/v). Peak identification and quantification (as weight percent) were made based on retention time and peak area of known standards (GLC 3C, 4C, 7C, 8C, 12C, and 20A; Nu Chek Prep, Inc., Elysian, MN, USA). The fatty acid concentrations are expressed as g per 100 g of the total identified fatty acids measured in each sample.

### 2.5 Statistical analysis

All data were analyzed by one‐way ANOVA using the GLM procedure of SAS (SAS Inst. Inc., Gary, NC). Statistical differences among treatments were tested by the Duncan's multiple range test. Results are presented as means ± *SD*. A *p*‐value of <0.05 was considered statistically significant.

## 3 RESULTS

### 3.1 Broiler performance

No mortality was observed during the trial. The performance of broilers in each treatment is shown in Table 3. During week 1–3, substitution of CSO for SBO had no significant difference in broiler performance compared with the control group (*p *>* *0.05). From week 4 to 6, 50% level of CSO inclusion significantly increased ADG and improved FCR compared with broilers in the control group (*p *<* *0.05). Similar results can be observed during week 1–6, and 50% level of CSO inclusion significantly increased ADG and improved FCR of broilers compared with the control group (*p *<* *0.05).

**Table 3:** Effects of dietary cottonseed oil on broiler performance^1^

Item	Levels of SBO replaced by CSO (%)				
	0	25	50	75	100
Week 1–3					
ADG (g)	43.4 ± 0.4^ab^	42.4 ± 1.7^b^	44.2 ± 0.8^a^	43.6 ± 0.8^a^	44.2 ± 0.6^a^
ADFI (g)	64.4 ± 0.5	64.6 ± 0.7	64.5 ± 0.7	64.0 ± 0.7	64.6 ± 0.4
FCR	1.49 ± 0.02^ab^	1.52 ± 0.07^a^	1.46 ± 0.04^b^	1.47 ± 0.03^b^	1.46 ± 0.02^b^
Week 4–6					
ADG (g)	81.9 ± 2.2^b^	82.5 ± 4.2^b^	86.0 ± 3.3^a^	82.5 ± 4.6^b^	83.9 ± 5.9^b^
ADFI (g)	145.4 ± 1.5	145.8 ± 4.3	145.6 ± 4.6	146.4 ± 5.4	147.4 ± 2.8
FCR	1.78 ± 0.06^a^	1.77 ± 0.06^a^	1.69 ± 0.04^b^	1.78 ± 0.05^a^	1.76 ± 0.10^a^
Week 1–6					
ADG (g)	62.6 ± 1.3^b^	62.4 ± 2.4^b^	65.0 ± 1.4^a^	64.0 ± 1.3^ab^	64.7 ± 2.2ab
ADFI (g)	104.9 ± 0.9	105.2 ± 2.4	105.0 ± 2.6	105.2 ± 2.6	106.0 ± 1.5
FCR	1.68 ± 0.04^a^	1.69 ± 0.05^a^	1.62 ± 0.03^b^	1.64 ± 0.05^ab^	1.64 ± 0.04^ab^

Notes

CSO: cottonseed oil; SBO: soybean oil.

1 Values are expressed as means ± *SD* (*n *=* *6), and means with different superscripts differ (*p *<* *0.05).

### 3.2 Serum biochemistry

The effects of inclusion of dietary CSO on serum biochemistry in broilers at week 6 are presented in Table 4. Compared to the control group, the serum activity of ALT in birds fed 25% CSO diets was increased (*p *<* *0.05), whereas the concentrations of TP, ALB, and CHOL were decreased with increasing CSO inclusion. Compared with broilers in the control group, high level of CSO (100%) reduced (*p *<* *0.05) the concentrations of TP, ALB, and CHOL, and the activity of AKP by 21.6, 21.0, 17.9, and 38.2%, respectively. In addition, the concentrations of ALB in birds fed 50% and 75% CSO diets were also significantly decreased (*p *<* *0.05) compared with the control group. However, substitution of CSO for SBO had no significant effects on serum activities of AST and GGT as well as the concentrations of HDL‐C and LDL‐C (*p *>* *0.05).

**Table 4:** Effects of dietary cottonseed oil on serum biochemical parameters in broilers at week 6^1^

Item	Levels of SBO replaced by CSO (%)				
	0	25	50	75	100
ALT (U/L)	5.1 ± 1.0^b^	7.4 ± 1.5^a^	3.9 ± 0.7^b^	4.6 ± 0.8^b^	5.5 ± 2.1^b^
AST (U/L)	554.5 ± 73.4^ab^	484.3 ± 64.71^ab^	366.0 ± 29.44^b^	648.7 ± 204.5^a^	694.0 ± 219.7^a^
TP (g/L)	38.5 ± 6.7^a^	35.4 ± 5.2^ab^	34.4 ± 3.6^ab^	33.2 ± 3.7^ab^	30.2 ± 1.8^b^
ALB (g/L)	16.24 ± 2.35^a^	15.22 ± 1.85^ab^	14.12 ± 1.35^bc^	13.85 ± 1.40^bc^	12.83 ± 1.06^c^
CHOL (mmol/L)	4.25 ± 0.39^a^	4.26 ± 0.56^a^	3.82 ± 0.50^ab^	3.84 ± 0.64^ab^	3.49 ± 0.24^b^
AKP (U/L)	1589.7 ± 71.5^a^	1560.9 ± 92.7^a^	1345.1 ± 97.5^ab^	1414.2 ± 115.9^ab^	982.8 ± 70.3^b^
GGT (U/L)	19.6 ± 6.6^ab^	24.0 ± 2.4^a^	23.1 ± 4.6^ab^	22.0 ± 4.1^ab^	17.2 ± 2.8^b^
HDL‐C (mmol/L)	2.23 ± 0.21^ab^	2.31 ± 0.30^a^	2.22 ± 0.16^ab^	2.10 ± 0.27^ab^	2.01 ± 0.06^b^
LDL‐C (mmol/L)	1.08 ± 0.24	1.05 ± 0.17	1.02 ± 0.18	0.97 ± 0.17	0.96 ± 0.15

Notes

AKP: alkaline phosphatase; ALB: albumin; ALT: alanine aminotransferase; AST: aspartate aminotransferase; CHOL: cholesterol; CSO: cottonseed oil; GGT: gamma‐glutamyl transpeptidase; HDL‐C: high‐density lipoprotein cholesterol; LDL‐C: low‐density lipoprotein cholesterol; SBO: soybean oil; TP: total protein.

1 Values are expressed as means ± *SD* (*n *=* *6), and means with different superscripts differ (*p *<* *0.05).

### 3.3 Serum antioxidant capacity

The effects of dietary CSO incorporation on serum antioxidant parameters are summarized in Table 5. At the end of the experiment, a numerical increase in the serum activity of T‐AOC was observed in high levels (75% and 100%) of CSO inclusion compared with the control group. Serum activities of GPX and SOD among all treatments were not affected. In addition, broilers fed 50%, 75%, and 100% CSO diets had a significant increase (*p *<* *0.05) in the activity of serum GST‐S compared with the control group. However, there was a numerical decrease in the MDA level for broilers fed 50%, 75%, and 100% CSO diets.

**Table 5:** Effects of dietary cottonseed oil on serum antioxidant parameters in broilers at week 6^1^

Item	Levels of SBO replaced by CSO (%)				
	0	25	50	75	100
T‐AOC (U/ml)	12.85 ± 2.28^ab^	9.54 ± 4.86^b^	14.80 ± 4.6^ab^	17.04 ± 5.13^a^	18.21 ± 3.68^a^
GPX (U/ml)	131.52 ± 49.24	169.80 ± 49.97	154.40 ± 54.63	143.80 ± 37.10	164.80 ± 42.85
GST‐S (U/L)	15.11 ± 4.58^d^	18.70 ± 4.54 ^cd^	24.52 ± 7.34^bc^	33.15 ± 2.74^a^	26.30 ± 3.97^c^
SOD (U/ml)	109.80 ± 4.46	110.11 ± 2.88	113.76 ± 1.77	107.36 ± 3.08	106.70 ± 10.04
MDA (nmol/ml)	3.23 ± 0.36^ab^	3.76 ± 0.26^a^	2.94 ± 0.47^a^	2.92 ± 0.61^b^	2.97 ± 0.65^b^

Notes

CSO: cottonseed oil; GPX: glutathione peroxides; GST‐S: glutathione S‐transferases; MDA: malondialdehyde; SBO: soybean oil; T‐AOC: total antioxidant capacity.

1 Values are expressed as means ± *SD* (*n *=* *6), and means with different superscripts differ (*p *<* *0.05).

### 3.4 Breast muscle fatty acid composition

The fatty acid profiles of breast muscle are shown in Table 6, and higher levels of CSO (50%, 75%, and 100%) significantly increased (*p *<* *0.05) the proportions of C14:0 and C18:0 compared with the control group. However, the proportions of C18:1n9t and C18:2n6c in high levels CSO (50 and 75%) diets were significantly decreased (*p *<* *0.05) compared to the control group. Birds fed 100% CSO had lower level of C20:2. In addition, there was a significant decrease (*p *<* *0.05) in the proportion of ∑ n‐6 PUFA for broilers fed higher levels of CSO diets. No significant difference was observed in the proportion of ∑ n‐3 PUFA among all the treatment groups.

**Table 6:** Effects of dietary cottonseed oil on fatty acid profile (g/100 g of total fatty acids) of breast muscle in broilers at week 6^1^

Item	Levels of SBO replaced by CSO (%)				
	0	25	50	75	100
C14:0	0.55 ± 0.04^b^	0.61 ± 0.03^ab^	0.59 ± 0.10^ab^	0.76 ± 0.15^a^	0.69 ± 0.11^a^
C14:1	0.05 ± 0.03	0.04 ± 0.04	0.06 ± 0.02	0.06 ± 0.04	0.04 ± 0.01
C15:0	0.11 ± 0.02	0.11 ± 0.02	0.12 ± 0.03	0.10 ± 0.01	0.09 ± 0.01
C16:0	32.83 ± 3.71	34.67 ± 2.30	34.97 ± 3.90	34.12 ± 4.32	34.84 ± 3.16
C17:0	0.20 ± 0.03	0.19 ± 0.02	0.22 ± 0.03	0.19 ± 0.03	0.18 ± 0.04
C18:0	15.29 ± 1.18^b^	15.31 ± 1.60^b^	18.77 ± 1.20^a^	18.45 ± 1.02^a^	18.93 ± 1.51^a^
C18:1n9t	3.90 ± 0.44^a^	4.19 ± 0.63^a^	3.48 ± 0.56^b^	3.84 ± 0.39^ab^	3.24 ± 0.70^b^
C18:1n9c	0.68 ± 0.03^ab^	0.70 ± 0.09^a^	0.58 ± 0.09^b^	0.60 ± 0.13^ab^	0.68 ± 0.12^ab^
C18:2n6c	0.97 ± 0.26^a^	0.91 ± 0.15^ab^	0.57 ± 0.11^c^	0.66 ± 0.11^bc^	0.76 ± 0.20^abc^
C20:0	0.18 ± 0.05	0.18 ± 0.03	0.19 ± 0.03	0.17 ± 0.03	0.19 ± 0.02
C18:3n6	0.99 ± 0.12^ab^	1.03 ± 0.13^ab^	1.06 ± 0.17^ab^	1.18 ± 0.25^a^	0.93 ± 0.14^b^
C18:3n3	0.16 ± 0.04	0.12 ± 0.06	0.13 ± 0.04	0.13 ± 0.04	0.12 ± 0.06
C20:2	15.29 ± 2.75^a^	15.14 ± 1.95^a^	13.11 ± 1.65^ab^	13.35 ± 1.09^ab^	12.14 ± 1.65^b^
C22:0	0.56 ± 0.15	0.29 ± 0.22	0.38 ± 0.25	0.32 ± 0.14	0.42 ± 0.23
C20:3n6	0.99 ± 0.12^ab^	1.03 ± 0.13^ab^	1.06 ± 0.17^ab^	1.18 ± 0.25^a^	0.93 ± 0.14^b^
C22:1n9	0.92 ± 0.36	0.80 ± 0.31	0.73 ± 0.38	0.75 ± 0.39	0.55 ± 0.13
C22:2	0.19 ± 0.02	0.17 ± 0.05	0.21 ± 0.05	0.21 ± 0.05	0.22 ± 0.08
C24:0	0.07 ± 0.03	0.04 ± 0.04	0.09 ± 0.03	0.07 ± 0.02	0.09 ± 0.05
∑n‐6 PUFA	2.15 ± 0.15^a^	1.92 ± 0.08^ab^	1.68 ± 0.09^bc^	1.61 ± 0.26^c^	1.66 ± 0.16^bc^

Notes

∑n‐6 PUFA: sum of C18:2n6c, C18:3n6, and C20:3n6; CSO: cottonseed oil; SBO: soybean oil.

1 Values are expressed as means ± *SD* (*n *=* *6), and means with different superscripts differ (*p *<* *0.05).

## 4 DISCUSSION

Due to the price fluctuates of the most commonly utilized raw materials in formulation of diets for monogastric animals, such as corn and soybean meal, it is urgent to find alternative ingredients that can substitute partially or totally of the conventional feedstuffs aiming at reducing the feed costs (de Sousa Lima et al., 2016). Cottonseed oil has been considered a potential substitute for SBO or corn oil in broiler diets because of their more accessible availability, similar metabolizable energy with SBO, and high concentrations of unsaturated fatty acids. In this study, weight gain, feed intake, and feed conversion ratio were not influenced by the level of inclusion of CSO during the starter period (week 1–3). It was because of low‐level oil implement in diet, as broilers had low requirement for energy during the starter period. During the finisher period (week 4–6), birds fed 50% level of CSO diet even had higher ADG and improved FCR compared broilers in the control group, which may due to the higher metabolizable energy content of CSO could meet the energy requirement of broilers (Rostagno et al., 2011). Similar results were reported by de Sousa Lima et al. (2016) who found that weight gain of broilers increased with the increasing CSO inclusion. However, a high rate of CSO replaced for SBO would increase the concentration of CPFAs in the diet, and high concentration of CPFAs may depress body weight gain. It was reported that *S*. foetida oil, which contained high concentration of sterculic acid, fed at moderate levels in the diet resulted in growth depression of weanling rats (Schneider, Sheehan, Vavich, & Kemmerer, 1968).

Elevated activities of ALT, AST, and GGT usually indicate hepatic injury (Sun et al., 2015). In the present study, serum AST and GGT activities were not affected by the substitution of CSO for SBO, except for low level (25%) of CSO increased serum ALT activity. In addition, reduction in the concentration of serum TP and ALB has also been reported to be sensitive serological indicators reflecting the synthetic function of the liver (Zeng et al., 2014). Serum concentrations of TP and ALB were reduced at 100% CSO level. The decrease in serum TP and ALB is an indication that CPFAs from CSO had negative effects on liver functions. Eisele et al. (1982) reported that male New Zealand weanling rabbits fed a diet containing 0.25% cyclopropenoid fatty acids for 28 days showed retarded growth and some moderate liver histological damage, when compared with the control ones. Nevertheless, the concentration of CPFAs in our study was much lower than previous studies, so it did not result in severe liver damage as previous studies. These results suggested that CSO may replace up to 50% of the SBO in diets for broilers without impairing the liver function. Given the comparatively high SFA and lower UFA profile of CSO, an atherogenic consequence may be expected with increased consumption of this oil. However, CSO has a lowering effect on CHO as demonstrated by previous studies (Davis, Prasad, & Imrhan, 2012; Radcliffe & Czajka‐Narins, 2006). In agreement with previous reports, we found that birds fed 100% CSO had lower CHO than the control broilers.

Lipid peroxidation substrates appear to impair the membrane function and endogenous antioxidant enzymes activity (Halliwell & Gutteridge, 2015) In our trial, higher serum T‐AOC activity and lower serum MDA content were observed in broilers fed high levels of CSO (50%, 75%, and 100%) diets, although the differences were not statistically significant. Additionally, significant increase in the activity of GST‐S in serum was found in response to high levels of CSO (50%, 75%, and 100%), which indicated that CSO could enhance antioxidant capacity. It may be due to relatively high antioxidant content in cottonseed oil, such as tocopherols (McLaughlin & Weihrauch, 1979; van Niekerk & Burger, 1985).

The breast muscle from the high levels of CSO (50%, 75%, and 100%) groups showed higher C14:0 and C18:0 proportion than the control group. It is well known that CPFAs in cottonseed oil are stearoyl‐CoA desaturase inhibitors, which can inhibit the conversion of stearic acid to oleic acid (Allen, Johnson, Fogerty, Pearson, & Shenstone, 1967; Evans, Davidson, LaRue, & Bandemer, 1963; Greenberg & Harris, 1982). Hence, saturated fatty acids increased in breast muscles of broilers fed CSO diet may be due to the presence of CPFAs in cottonseed oil. Although public concerns about SFA in meat, C18:0 might not affect CHO concentrations in human blood (Simopoulos, 2001). Only C14:0 and C16:0 appear to have hypercholesterolemic properties (Daley, Abbott, Doyle, Nader, & Larson, 2010). The C14:0 proportion in the high levels of CSO (75% and 100%) groups was significantly higher than that of the control group, suggesting that using high levels of CSO may have a negative impact in fatty acid profile with respect to human health. The most striking difference between the fatty acid profile of SBO and CSO is the lower level of C18:1n9t and C18:3n3 in CSO. This difference was responsible for the lower values for C18:1n9t and C20:2 in breast muscle. This indicates that CSO is not suitable as SBO as a source of C18:1n9t. N‐3 PUFAs play an important role in prevention of cardiovascular disease (Simopoulos, 1999). In the present study, the n‐3 PUFA was not different among the experimental groups. Among the ∑ n‐6 PUFAs, C18:2n‐6 is an important fatty acid, which is the precursor of arachidonic acid (Ebrahim et al., 2015). Arachidonic acid is crucial for the production of eicosanoids, including prostaglandins, thromboxanes, and leukotrienes (Bourre et al., 1993). Based on the results of this study, high levels of CSO decreased the amount of C18:2n‐6 and ∑ n‐6 PUFAs in the breast muscle of broilers, which was different from the result of Rostagno et al. (2011), who found the use of CSO had no effect on C18:2n‐6 and ∑ n‐6 PUFAs status in adipose lipids of mice. This discrepancy may be caused by differences in animal species and sampling sites.

## 5 CONCLUSIONS

In conclusion, CSO containing CPFAs (0.20%) may replace up to 50% of the SBO in diets for broilers without impairing the performance, liver functions, and breast muscle fatty acid composition of these animals after CSO having been refined and processed. These findings indicated that refined CSO could be used as a potential substitute for SBO in poultry production.

## CONFLICT OF INTEREST

The authors declare that they do not have any conflict of interest.

## ETHICAL STATEMENT

The experimental animal procedure was approved by the Scientific Ethics Committee of Huazhong Agricultural University.
